# Assessing the Impact of an Intervention Project by the Young women's Christian Association of Malawi on Psychosocial Well-Being of Adolescent Mothers and Their Children in Malawi

**DOI:** 10.3389/fpubh.2021.585517

**Published:** 2021-03-24

**Authors:** Mtisunge Kachingwe, Ibrahim Chikowe, Lotte van der Haar, Nettie Dzabala

**Affiliations:** ^1^Young Women's Christian Association of Malawi, Blantyre, Malawi; ^2^Queen Elizabeth Central Hospital, Ministry of Health Malawi, Blantyre, Malawi; ^3^Pharmacy Department, College of Medicine, University of Malawi, Blantyre, Malawi; ^4^Utrecht Centre for Global Challenges, Utrecht School of Economics, Utrecht University, Utrecht, Netherlands

**Keywords:** adolescent motherhood, psychosocial well-being, early childhood development, mental health, NGO program evaluation

## Abstract

Adolescent mothers in Malawi face psychosocial challenges such as low resilience level, low self-esteem, poor maternal-infant interaction, and exposure to intimate partner violence (IPV). Children of adolescent mothers often face numerous risks such as low birth-weight, stunted growth, infant death, low school enrolment, increased grade repetition, and dropouts that put them at greater risk of poor developmental outcomes and socio-emotional problems. This study assessed the impact of components of a community project conducted by the Young Women's Christian association of Malawi in providing psychosocial support to adolescent mothers and their children. The goals of the project were; (1) to improve early childhood development in babies born to adolescent mothers; and (2) to enhance the psychosocial well-being of adolescent mothers (self-esteem, resilience stress, and parenting skills). This descriptive mixed methods evaluation study comprised an intervention and control groups of adolescent mothers respectively. The project had 3 centers in southern region districts of Malawi. Target population was adolescent mothers 18 years of age and below. At baseline we enrolled 267 mothers and at the end of the project we had 211 mothers. The project involved monthly meetings with adolescent mothers imparting knowledge and skills and early childhood education activities. From July 2017 to June 2019, 58 sessions were conducted. In the first year the control group had no meetings, however they received the intervention in the second year. Overall results in the intervention group showed statistically significant increase in knowledge on parenting skills (*p* < 0.01), nutritional practice (*p* < 0.01), motor skills and cognitive functions in children (*p* < 0.01) as well as expressive language and socio-emotional capacities in children (*p* < 0.01), while the change in confidence and psychosocial well-being was not statistically significant (*p* = 0.8823). Community projects such as these enhance parenting skills and improve development of children born to adolescent mothers. Improving psychosocial support is complex and requires further research and a more holistic approach.

## Introduction

Malawi has one of the highest rates of teenage pregnancy rates, currently at 29% of the population and has a population of about 17.5 million (1, 2). Adolescent mothers give birth, care, and provide for their children while they are still children themselves and lack practical child rearing skills (3, 4). Children raised in households of adolescent mothers are more at risk of low birth-weight, stunted growth, infant death, low school enrolment, increased grade repetition, and dropout (5–7). In Malawi, babies born to women with no education experience an under 5 mortality rate of 138 per 1,000 live births, in comparison to 94 per 1,000 for women with secondary education. The prevalence of stunting is 12% higher for children born of adolescent mothers than of mothers over the age of 20. Adolescent parenthood is associated with mental health problems such as depression, substance abuse, and posttraumatic stress disorder (8). In addition to this, adolescent mothers have often dropped out of school and thus have less information about prevention of mother to child transmission, health services which infants need in the first 1,000 days, parenting, nutrition, childcare, and stimulation (4, 9, 10).

Various programs have been implemented in low income countries aimed at preventing teenage pregnancies, marriage, and sexually transmitted infections (11–15). However, an area that is lacking includes interventions aimed at supporting married or single adolescent girls who are pregnant or are already mothers together with their children. Programs and centers focusing on psychosocial support are valuable in addressing the holistic needs of children, teenage mothers and their families (16, 17). Use of psychosocial support in early childhood development centers enhances the centers to not only provide educational enrichment, nutrition, healthcare and safety, but also help children and their caregivers to grow a sense of self-worth, participation, social connectedness, and full enjoyment of life. It also helps build better connections between children and their care givers (8, 17).

The Young Women's Christian Association of Malawi (YWCA MW) conducted an intervention project in rural communities of three districts (Mulanje, Blantyre, and Machinga) in southern Malawi titled “Community model for fostering health and well-being amongst adolescent mothers and their children.” The goals were (1) to improve early child development in babies born to adolescent mothers, (2) to enhance the psychosocial well-being of adolescent mothers (self-esteem and resilience) and (3) to assess the impact of interventions on the young mothers and the children.

## Materials and Methods

### Study Design and Participants

This was a descriptive mixed methods evaluation study for the project community model for fostering health and well-being amongst adolescent mothers and their children. The project was conducted in 3 centers, with each center being located in each of the 3 districts in southern region of Malawi namely Mulanje, Blantyre, and Machinga. The sites were chosen due to the fact that the YWCA has branches in these areas. Target population was adolescent mothers 18 years of age and below who voluntarily agreed to participate after an informed consent. Written informed consent to participate in this study was provided by the participants or their legal guardian/next of kin.

Various methods were used to identify and recruit the mothers into the project (1) antenatal clinics, (2) community leaders, (3) social workers and peer recommendation. At baseline, we recruited 267 mothers and their children and at the end of the project we had 211 mothers (56 dropouts). The dropouts cited several reasons for dropping out, which included continuing education, employment, business, marriage, or relocation from the project site.

The adolescent mothers were divided into two groups of intervention, namely intervention and control groups, and the division process was based on random numbers. The intervention group received the training in various aspects of psychosocial life while the control group did not receive training. But after 1 year, the control group commenced to receive the intervention and the two groups run parallel sessions simultaneously. This set up allowed us to (1) make a comparison between treatment and control for the first year, and (2) to allow comparison if duration of intervention would impact results (a 1-year intervention vs. a 2 year intervention).

### The Intervention

Various steps were taken to inform project design and intervention program namely meetings with stakeholders (government ministries, village leaders, other NGOs) and a baseline study/needs assessment was conducted (Dzabala et al., unpublished).

The intervention comprised provision of safe and inclusive community spaces for adolescent mothers and pregnant girls. YWCA coordinators and champions held two dialogue sessions a month at YWCA safe spaces in Mulanje, Blantyre, and Machinga and each session lasted 4 h. The facilitators were monitored for quality by the project officer, project manager and executive director. In some sessions key resource personnel were invited to facilitate sessions, e.g., social workers and health workers.

Each district set up a team comprising 5 YWCA champions plus district coordinator. Champions were YWCA peer educators and members who were trained to facilitate the meetings with adolescent mothers and their children. From July 2017 to June 2019 a total of 58 sessions were conducted (36 sessions for intervention group and 22 sessions for control group). On average each adolescent mother and child attended at least 80% of the sessions.

Psychosocial programs were used to build resilience, self-esteem, and mutual support among girls. The program aimed at empowering girls to prioritize their own and their children's health and well-being by providing them with services and information. The sessions were not designed as lectures but applied principles of adult learning, offered craft activities, small group talks, and repeated opportunities to practice positive parenting. Table 1 provides an overview of the intervention components, beneficiaries, and deliverers of the community program.

**Table 1:** Components, beneficiaries, and deliverers of the adolescent mothers project.

**Intervention components**	**Description**	**Beneficiaries**	**Deliverers**	**Number of sessions**
Early childhood development and stimulation	Early childhood education and development including s*timulation of babies with toys to help development of cognitive function, Track health passports for children to monitor health visits, immunizations, growth, and development milestones*	Children born to adolescent mothers	Community based YWCA volunteers and staff early childhood coordinators	56
Teenage mothers support group	Meetings twice a month where Received information of the following topics information and services on topics such as; *Prenatal care, nutrition, antenatal and postnatal visits, healthy child delivery, child health, and services; Sexual Reproductive Health Rights (SRHR), including Sexually Transmitted Infections (STIs), Family Planning (FP), access to contraception, Gender based violence and Gender equality*; *Parenting skills—health care, nutrition, breastfeeding, child care, child safety, stimulation, and development (infants to be brought to all sessions)*	Teenage mothers	Community based YWCA volunteers and staff Health workers Social workers	56
Community Awareness raising during community meetings	Awareness during community meeting *Engage health care workers in* *Engage stakeholders*	Community members National and district leaders	YWCA staff and volunteers	15
Counseling	Aimed at coping with stigma, reconciliation of relationships between parents and teenage mothers, and advice for continued education.			50
Livelihood and health services	Linkages with Back to school programs *linkages/referrals to health Links to livelihood programs Links to school re-entry programs*	Adolescent mothers		48

### Data Collection

Data collection was done by officers from the district social welfare office of the Ministry of Gender, Children, Disability, and Social Welfare based in the study district. This is the ministry that implements early childhood development programs in Malawi. Fifteen data collectors were identified, five from each district. The data collectors were recruited based on their previous involvement and experience with adolescents and young mothers and these included 7 social welfare officers, 2 assistant social welfare officers, 5 child protection officers, and 1 Early Childhood Development (ECD) personnel. The data collectors received training consisting of theory and field trial prior to commencement of data collection.

Data was collected through interviews, direct observation of parent-child interactions, developmental assessments, and interviews of adolescent mothers using a structured questionnaire. The questionnaires for the interviews and checklists for developmental milestones were developed by adapting questionnaires used in similar projects. The questionnaire(s) were pretested prior to data collection exercise to ensure that they were still valid after the adaptation and this was done by interviewing young mothers outside the project areas and evaluating the answers for correctness. The questionnaires and checklists were then installed in mobile tablets for easy data collection and collation. The interviews were performed at the YWCA branches away from their homes and spouses. Each interview and/or observation took ~30–45 min to complete. Data collectors were supervised at least once during the data collection period by the project officer.

The questionnaire had two parts. The first part collected data on the developmental milestones (nutrition status and child development) and physical growth of the children. A 100% score on these indicators means that the child met all the age-relevant developmental milestones. Based on the responses, we were able to create a score for the relevant age group and then convert this to a percentage score, which allowed for analysis across age groups.

The second part of the questionnaire comprised tools to assess the psychosocial well-being and parenting skills of mothers. We measured resilience through the Brief Resilience Scale (18), Self-esteem was measured using the Rosenberg Self-Esteem Scale) (19). Parental stress was measured using the Parental Stress Scale (20). And mother infant interaction was assessed using a tool adapted from the assessment of Mother Infant Sensitivity (AMIS) Scale (21).

Qualitative data on these indicators was also collected through semi-structured interviews. This aimed to add richness and more information to the quantitative data. It explored the effects of the project on the social environment and on the psychological and social well-being of adolescent mothers. Forty one individual semi-structured interviews conducted with: 18 beneficiaries (adolescent or young mothers); 18 peer champions who ran the groups; 2 project staff members and 3 trainers. 41 individual semi-structured interviews conducted with: 18 beneficiaries (adolescent or young mothers); 18 peer champions who ran the groups; 2 project staff members and 3 trainers. Respondents were asked about their experiences on the project. The questions explored the following broad areas; impact of project on beneficiaries and challenges faced; case studies; challenges with the project; changes observed in beneficiary children; impact of project on peer champions and project staff; most useful parts of the project; and recommendations and areas of improvement.

### Measurements Time-Points and Measures of Evaluation

Data about the mothers and their children was systematically collected at before the interventions (baseline) and after the implementation of the project (endline). To assess the impact of participating in the intervention, both the quantitative measures and qualitative data mentioned above were used. The study had five outcomes namely (1) increased knowledge on parenting skills, (2) increased knowledge on nutritional practices, (3) improved confidence and psycho-social well-being, (4) improved motor skills, cognitive functions, and (5) improved expressive language and socio-emotional capacities. Table 2 highlights the measurement tools and indicators used to measure outcomes.

**Table 2:** Project outcomes and the activities or measures used to achieve them.

**Outcome**	**Measures**
Psychosocial domain	
Increased knowledge on parenting skills	Mother-infant interaction score
Increased knowledge on nutritional practices	Number of Children with Improved Physical Growth as indicated by: Length/height-for-age Weight-for-age Weight-for-length/height Body mass index-for-age (BMI-for-age) Head circumference-for-age Motor development milestones reached
Improved confidence and psycho-social well-being	Resiliency levels of mothers Self-esteem levels of mothers Parental Stress levels of mothers
Improved motor skills, cognitive functions	Motor and cognitive development milestones reached shown by: Number of children with improved development as indicated by improvements in various indicators under the following dimensions: Learning, thinking, and problem-solving.
Improved expressive language and socio-emotional capacities	Number of Children with Improved Development in Language and Communication as indicated by improvements in various indicators under the following dimensions: - Hearing and Understanding - Talking Number of Children with Improved Social and Emotional Development as indicated by improvements in various indicators under the following dimensions: - Interaction with others - Emotional regulation - Emotional responsiveness

### Data Analysis

Quantitative data was imported into STATA v14 for statistical analysis. Data was cleaned and also entailed coding and encoding. Entries with missing data were excluded from the analysis. Missing entries that were excluded were those of respondents that dropped out and had no data at all. Both descriptive and bivariate statistics were conducted to examine the distribution of all variables and assess relationships between variables. Collected data elements on various developmental measurement scales were converted into scores using the particular standard scoring methods. Following the scoring the measuring scales components, the values of particular scale components were summed up to come up with a total score of the outcome measure. We compared the mean score between the intervention and control groups at baseline and endline.

*t*-test models were used to assess the effectiveness of the intervention comparing mothers and children belonging to the two groups. A treatment effect analysis was done to compare the outcomes at the end of 2 years in the intervention group compared to control group. A *P*-value of 0.05 was used as a measure of significance.

The qualitative interviews were audio recorded, followed by detailed notes with quotes for each interview were written up by the data collector. These were then imported into N-Vivo, a qualitative data analysis software program. Each response was analyzed using thematic content analysis. In this technique, each response is coded into themes. The themes were then cleaned by joining some together and creating broader themes. During this process, important quotes and case studies illustrating themes were noted.

## Results

### Demographics

Two hundred and eleven mothers were evaluated. Average age of participants was 17.56. 49.3% (104) of the mothers were married and living with the husband. 12.8% of the mothers lived alone with their children and had no support structures. The rest of the participants had support structures in the form husband, father/mother, grandparents, siblings, extended family, and in laws. Demographic data of participant in summarized in Table 3. Majority of the mothers (188, 89.43%) had given birth to one child, while 22 (10.4%) had given birth to more than one. Only one respondent reported having given birth to three children. None of the respondents were pregnant at the time of recruitment. Ages of the children that were recruited to be followed throughout the project ranged from 1 to 36 months (*M* = 12.79 months, SD = 7.45). The sex of the children was almost equally divided between female (52.06%) and male (47.94%).

**Table 3:** Demographic and demographic data of the study participants.

**Parameter**	**Variable**	**Number of mothers (*n* = 211)**	**Percentage (%)**
Respondents	Mulanje	74	35.1
	Blantyre	69	32.7
	Machinga	68	32.2
Age	13–18	183	86.7
	19–22	28	13.3
Marital status	Married/domestic partnership	130	61.61
	Single/never married	25	11.8
	Separated	32	15.2
	Having boyfriend/not living together	18	8.5
	Divorced	6	2.8
Type of household	Hut made of traditional materials	103	48.8
	House made of bricks or concrete	103	48.8
	Other	1	0.5
	Living on the street	1	0.5
	Boys quarters	3	1.4
Person living with the young mother	Husband	104	49.3
	Parents	36	17.1
	Grandparents	16	7.6
	Alone/alone with child	27	12.8
	Siblings	7	3.3
	Extended own family	15	7.1
	Mother/father in law	6	2.8
Type of support given by child's father^*^	Material support	155	73.5
	Financial	142	67.3
	None	83	39.3
	Social	83	39.3
	Emotional	62	29.4
Education level^*^	Primary Education	134	63.5
	Secondary education	77	36.5

* *Multiple responses were possible therefore percentages add up to more than 100*.

### Quantitative Outcomes on Children

Quantitative outcomes explored nutrition, physical growth, and developments milestones of the children. Nutritional improvements in both the control and intervention groups are showed in Table 4. There were improvements for “Most meals were baked, steamed or boiled” (so not fried), ranging from 39.6% to 71.6% in the intervention group. Similar results were seen in the control group with improvements from 43.7% at baseline to 64.8 at endline.

**Table 4:** Nutritional improvement scores for the children of young mothers.

	**Intervention group**				**Control group**			
	**Baseline (*****N*** **=** **134)**		**Endline (*****N*** **=** **106)**		**Baseline (*****N*** **=** **135)**		**Endline (*****N*** **=** **105)**	
	**Yes**	**%**			**Yes**	**%**	**Yes**	**%**
Child eats raw vegetables and fruits as snacks instead of snacks that are high in sugars or fat	50	37.31%	67	63.21%	61	45.19%	63	60.00%
Most meals are baked, steamed or boiled (so not fried)	53	39.55%	75	70.75%	59	43.70%	68	64.76%
Child eats more chicken and fish than red meat in a week	31	23.13%	76	71.70%	34	25.19%	67	63.81%
Child does not drink cool drinks (high in sugar)	22	16.42%	41	38.68%	25	18.52%	45.00	42.86%
Child eats different kinds of fruits and vegetables in a week	62	44.94%	95.00	89.62%	58	42.96%	92.00	87.62%

On physical growth there were improvements on physical growth indicators of children between baseline and endline period mostly for the unpleasant ‘'below expected (below the 15th percentile)” category in both groups (Table 5). At baseline, most children (66.1% in the intervention group and 64.9% in the control group) fell within the expected levels of physical development (between 15th and 85th percentile^1^) across all indicators. At end line, there were improvements for length, head circumference, while weight, weight for length) and BMI had larger improvements for the proportion of children that were below expected. Table 5 below summarizes the physical growth indicators.

**Table 5:** Physical growth indicators.

	**Intervention group**						**Control Group**					
	**Baseline (*****N*** **=** **134)**			**Endline (*****N*** **=** **106)**			**Baseline (*****N*** **=** **135)**			**Endline (*****N*** **=** **105)**		
**Area assessed**	**Below expected (below the 15th percentile)**	**As expected (between 15th and 85th percentile)**	**Above expected (above 85th percentile)**	**Below expected (below the 15th percentile)**	**As expected (between 15th and 85th percentile)**	**Above expected (above 85th percentile)**	**Below expected (below the 15th percentile)**	**As expected (between 15th and 85th percentile)**	**Above expected (above 85th percentile)**	**Below expected (below the 15th percentile)**	**As expected (between 15th and 85th percentile)**	**Above expected (above 85th percentile)**
Length	29 (21.6%)	86 (64.2%)	19 (14.2%)	20 9(18.9%)	74 (69.0%)	12 (11.3%)	42 (31.1%)	87 (64.4%)	6 (4.4%)	34 (32.1%)	63 (59.4%)	8 (7.5%)
Weight	23 (17.2%)	96 (71.6%)	15 (11.2%)	9 (8.5%)	85 (80.2%)	12 (11.3%)	25 (18.5%)	96 (71.1%)	14 (14.8%)	10 (9.4%)	89 (84.0%)	6 (5.7%)
Weight for length	13 (9.7%)	89 (66.4%)	32 (23.9%)	3 (2.8%)	57 (53.8%)	46 (43.4%)	20 (14.8%)	87 (64.4%)	28 (20.7%)	2 (1.9%)	53 (50.0%)	50 (47.2%)
BMI	28 (20.9%)	78 (58.2%)	28 (20.9%)	7 (6.6%)	64 (60.4%)	35 (33.0%)	31 (23.0%)	77 (57.0%)	27 (20.0%)	7 (6.6%)	53 (50.0%)	45 (42.5%)
Head circumference	8 (6.0%)	94 (70.1%)	32 (23.9%)	7 (6.6%)	42 (39.6%)	57 (53.8%)	17 (12.6%)	91 (67.4%)	27 (20.0%)	8 (7.5%)	47 (44.3%)	50 (27.2%)
**Average %**	**15.08%**	**66.10%**	**18.82%**	**8.68%**	**60.60%**	**30.56%**	**20.00%**	**64.86%**	**15.98%**	**11.50%**	**57.54%**	**26.02%**

There were also improvements of the motor, cognitive, social and emotional, language, and communication scores of children (summarized in Table 6).

**Table 6:** Proportion of children with improved outcomes.

		**Proportion of children with improved outcomes in the intervention group *N* = 107 (%)**	**Proportion of children with improved outcomes in the control group *N* = 96 (%)**
Improved outcomes for children served: child growth and developmental status	Children with improved physical growth	104 (97%)	89 (93%)
	Children with improved cognitive development	103 (96.2)	80 (83%)
	Children with improved development in language/ communication	102 (95%)	83 (86%)
	Children with improved social and emotional development	107 (100%)	91 (95%)
Improved outcomes for children served: health and nutrition	Children with improved child nutrition and diet	103 (96%)	91 (95%)

### Psychosocial Well-Being of Young Mothers

#### Resilience

74.39% of mothers agreed or strongly agreed that they tend to bounce back quickly after hard times at baseline and this increased to 92.42% at endline. 66.18% agreed or strongly agreed that it does not take them long to recover from a stressful event at baseline and this increased to 78.67% at endline. However, 65.75% agreed or strongly agreed that it is hard for them to snap back when something bad happens and 63.77% agreed or strongly agreed that they have a hard time making it through stressful events at baseline. At endline, these parameters increased to over 71.09 and 78.20%, respectively (Supplementary Table 1).

#### Self-Esteem

Almost all of the mothers (93.72%) agreed or strongly agreed that they take a positive attitude toward themselves and this increased slightly at endline to 96.79%, while 87.44% agreed or strongly agreed that on the whole, they were satisfied with themselves and this increased to 90.05% at endline. However, several mothers agreed or strongly agreed that they wish they could have more respect for themselves (96.62%) but this decreased slightly at endline to 95.73; that they do not have much to be proud of (71.49%) and this increased to 72.04% at endline and 57.00% that they at times think they are no good at all at baseline and saw a slight increase at endline to 58.77% (Supplementary Table 2).

#### Parental Stress

Over 80% of mothers agreed or strongly agreed that: they were happy in their role as a parent at baseline and improved to 94.79%; they feel close to their child; they enjoy spending time with their child; their child is an important source of affection for them; having a child gives them a more certain and optimistic view for the future; and they find their children pleasurable. On the other hand, some mothers agreed or strongly agreed that they sometimes worry whether they are doing enough for their child (73.43%) at baseline with slight increase at endline to 78.20%, that caring for their child sometimes takes more time and energy than they have to give (63.29%) at baseline to 67.67% at endline, that having a child has meant having too few choices and too little control over their life (63.34) at baseline which decreased to 58.77% at endline. Finally, 42.03% of mothers agreed or strongly agreed that if they had to do it over again, they might decide not to have a child at baseline and it dropped to 29.38% at endline (Supplementary Table 3).

#### Mother-Infant Interaction

The Mother-Infant Interaction Score ranges from 5 to 25 with a higher score indicating increased positive mother-infant interaction (21). At baseline, most mothers' (37.56%) predominant maternal mood/affect was characterized by “shifts of expression in response to Infant behavior such as prolonged visual regard and smiling at Infant”. At endline, this increased to 82.94%. Improvements in the mother-child interaction were also observed in other aspects of mother-child interaction (Supplementary Table 4).

#### Intimate Partner Violence

Before asking respondents about their exposure to IPV, they were asked to indicate how safe they felt in their homes and in their community. At baseline, most felt safe or completely safe in their home (73.41%) and in their community (75.28%), but at endline this improved to 90.04 and 92.4%, respectively. Most of the mothers (89.51%) reported that they have been in an intimate relationship. 61.92% said that they were currently in an intimate relationship. Of these, 31.08% said that they were currently afraid of their partner. The IPV scale assessed the exposure of the mother and the child to 15 forms of IPV in the last 3 months. The forms of IPV reported by the highest number of mothers were: “My partner told me I was crazy, stupid or not good enough” (24.53%) at baseline but reduced to 11.85% at endline; “My partner blamed me for causing their violent behavior” (21.05%) at baseline which reduced to 9.95% at endline; “My partner made me perform sex acts that I did not want to perform” (17.11%) at baseline that reduced to 10.95% at endline; and “My partner shook, pushed, grabbed or threw me” (17.05%) at baseline that reduced to 11.85% at endline (Supplementary Table 5).

### Impact of Interventions on the Study Outcome: Relationships Between Variables

On the part of the intervention group, there was statistically significant increase in knowledge on parenting skills (*p* < 0.01), nutritional practice (*p* < 0.01), motor skills and cognitive functions (*p* < 0.01) as well as expressive language and socio-emotional capacities (*p* < 0.01), while the change in confidence and psychosocial well-being was not significant (*p* = 0.8823), showing that there was no change. However, there was no significant change for the control group for all parameters. Table 7 shows control group and intervention groups that had significant and insignificant changes during the study period.

**Table 7:** Outcomes at 2 years compared to baseline in the intervention and control group.

	**Intervention group**					**Control group**				
	**Baseline mean**	**Endline mean**	**Effect size mean difference**	**95%CI**	***p*-value**	**Baseline**	**Endline**	**Effect size**	**95%CI**	***p*-value**
Outcome 1 Increased knowledge on parenting skills	17.19	18.65	1.46	(2.05, 0.87)	<0.0001	17.87	17.94	0.068	(0.541, 0.676)	0.827
Outcome 2 Increased knowledge on nutritional practices	7.56	10.84	3.27	(3.89, 2.65)	<0.0001	8.98	9.03	0.046	(0.632, 0.724)	0.894
Outcome 3 Improved confidence and psycho-social well-being	94.20	94.29	0.098	(−1.39, 1.20)	0.8823	94.15	94.34	0.188	(1.109, 1.486)	0.776
Outcome 4 Improved motor skills, cognitive functions	17.31	39.22	21.91	(23.79, 20.03)	<0.0001	26.76	27.12	0.363	(2.339, 3.064)	0.792
Outcome 5 Improved expressive language and socio-emotional capacities	31.71	78.94	47.23	(52.42, 42.05)	<0.0001	54.19	50.75	−3.438	(10.083, 3.208)	0.310

### Qualitative Results

#### Most Useful Parts of the Project

From the education sessions point of view, the topics on brain development was ranked as the most useful part (by 71% of all respondents), followed by how to care for and protect your child (51%), relationship building (34%), and stress management (32%).

#### Impact of the Project on the Adolescent Mothers and Their Children

Respondents were asked to reflect on what the impact of the project has been on the adolescent and young mothers. Responses included that they have become better mothers (90% of all respondents), have experienced a personal change or development (83%), feel that they have improved health and hygiene (28%), have improved relationship building (56%), and the project allowed access to other opportunities (17%).

Respondents were asked if they noticed any changes in the children of the adolescent and young mothers as a result of the project. Four themes emerged from these responses, namely physical and intellectual growth (94%), improved eating or diet (56%), improved social skills and confidence (72%), and improved health (33%). One beneficiary recalls the impact of the project on her child.

A treatment effect analysis was conducted to compare the outcomes at the end of 2 years in the intervention group compared to control group. The results showed 17.9-point increase in the average score in the intervention group compared the control group at the end of 2 years follow up. However, this increase was not statistically significant 17.9 95% CI (−3.23 to 39.11), *p*-value 0.097.

## Discussion

The results of the intervention evaluation suggest that the intervention programs were effective in dealing with the challenges that young mothers and their children faced. The study had five outcomes namely (1) increased knowledge on parenting skills, (2) increased knowledge on nutritional practices, (3) improved confidence and psycho-social well-being, (4) improved motor skills, cognitive functions and (5) improved expressive language and socio-emotional capacities. This was shown by comparing outcomes at baseline and endline, for both the intervention and control groups. There was a significant change in the four of the five outcomes that were evaluated for the intervention groups (Table 7). Notably, 79% (211/267) of the young mothers participated in the intervention programs for 2 years. The retention rate was good for such a project, though it was slightly lower for similar intervention program (91%) in Canada that was done in a minimum of six weeks sessions as compared to ours that took 2 years. But our retention level was higher than many similar programs in the US that mostly have 40–60% drop out rate (17). We attribute this high retention rate to the study design approach. As an incentive the control group received the intervention in the second year. This gave them something to look forward to while maintaining integrity of the program to have comparison group. In addition to this the project involved the community and tailored the programs for the needs of the targeted people (Dzabala et al., unpublished). We also attribute the retention rate to the interest that the program brought to the participants and the challenges it solved for them as cited in the semi-structured interviews. Another contributing factor was that the sessions were run by trained YWCA Malawi members. These reside right in the communities and were known to the participants hence security of safe space and absentees were followed up if they missed two consecutive sessions.

Generally, the project resulted in increased knowledge on parenting skills and nutritional practices. This suggests that the materials, methods, personnel, and duration that the interventions were implemented were effective for the purpose. This is in agreement with the literature that shows that awareness of the mothers improves their preparedness in performing some tasks, like in this case parenting and nutritional practices (22).

Analysis in the control group showed no statistical difference in all outcomes measures at the end of 2 years (Table 7). One possible reason for this was that the study group was underpowered. Majority of study dropouts were in the control groups therefore reducing the sample size. Secondly this could alo be due to the fact that the control group received the intervention for shorter duration. Tintervention groups received 36 sessions and control receiving 22 sessions. This difference may suggest that duration of intervention and number of sessions matters in the effectiveness of the intervention. This implies that in the implementation of such intervention, time should be considered factors when programming. This contradicts a similar study in Nigeria whose results implied that modest levels of participation could enable community uptake of a potentially lifesaving health intervention as well as promotion of policy change (23). The 1 year of intervention the control group received may have not been enough time to learn and start practicing what they learnt. Therefore, in order to achieve the same results in shorter period, curriculum review of the program may need revision, either in the delivery approach or content being taught. This may be supported by studies that have shown that parenting is multidimensional. This means that parents need more knowledge to meet children's needs. This knowledge should be both deep and wide enough that includes developmental milestones and norms that support parenting (24).

Overall results in the intervention group showed that there was significant improvement in the, children's motor skills and cognitive functions as well as improved expressive language and socio-emotional capacities (Table 7). This suggests that the intervention programs were effective for the children as well. Children's physical health, social, cognitive, emotional, and behavioral competences are suggested to be contributed by the parents' knowledge, attitudes and practices. Children's outcomes depend on various domains of development, which in turn are also enhanced by early positive and supportive interactions with parents or caregivers. For young children to thrive and have proper growth in all life domains, they need their parents and other primary caregivers, inside and outside the home (25) Once the physical health and safety of the children in achieved, it helps them to achieve the other domains such as emotional and behavioral competence, social competence, Cognitive and Competence (25, 26). This therefore also means that the programs that target different domains of life are more successful. For future program we recommend such holistic approaches incorporating different domains.

For both the intervention and control group the outcome measure improved confidence and psychosocial well-being was not statistically significant (Table 7). The insignificant change could be due to the multidimensional state of psychosocial well-being that includes many factors that are also interrelated. There are many domains of psychosocial well-being such as happiness, satisfaction with life, positive effect, optimism, hope, vitality, purpose in life, personal growth, self-acceptance, environmental mastery, autonomy among others (27) yet only fewer of those domains were considered in this study (Table 2). In this cohort most mothers struggled with purpose in life, with majority being school dropouts and living in poverty. This is an area that the project did not address. Therefore, the insignificant result could be due to failure in the programs to improve one sector of the psychosocial well-being the affected the achievement of the other domains significantly. Improvement in one dimension of psychosocial also depends on the presence of other psychosocial parameters such that designing of such programs should always consider incorporating all psychosocial well-being dimensions (28). Policies which seek to enhance the psychosocial well-being of adolescent mothers should aim to do this through existing services.

### Limitations of the Study

The findings should be interpreted within the limitations of the study. Study participants were selected based on convenience; the confounding effects were not controlled during the study design or at the end of the project using statistical measures. This made it challenging to look at the overall impact of the intervention statistically. Therefore, the results cannot be generalized and establish a cause and effect pattern. There are limitations with convenience sampling however, it was used in this study because the intervention was a pilot in the communities, this approach was cost effective and the nature of the study it was challenging to create constant variables in the communities for adequate comparison. In addition, the tools used in this study have not been validated in Malawi and contextual different might have had an impact on their suitability to assess the parameters and should be interpreted with caution. The questionnaire was pretested on a separate population and amendments to the questionnaire were made where necessary.

The study also did not include a comparison group of adolescents who have not given birth and who are not pregnant to assess the degree to which the pregnancy/child affected psychosocial well-being. Despite these limitations, this study provides insight into the experiences of a vulnerable group for which little information exists in terms of psychosocial well-being. It also provides useful information for interventions aimed at this group.

## Conclusions

This study confirms the widely reported results elsewhere that young mothers face many psychosocial challenges and underscores the need for projects targeting this group of vulnerable mothers and children. It further highlights the need to understand and enhance the psychosocial well-being of adolescent mothers. We found that our intervention, targeted at improving the psychosocial support of adolescent mothers, increased child development but did not increase the mothers' psychosocial well-being. While policy makers can use this intervention to increase child development of adolescent mothers, future iterations of this work should focus on increasing (our understanding of) the psychosocial wellbeing of the mothers.

## Data Availability Statement

The datasets presented in this article are not readily available because the data set is not anonymized and consent was not obtained to share with third parties. Requests to access the datasets should be directed to Mtisunge Kachingwe, mtkachingwe@gmail.com.

## Ethics Statement

The studies involving human participants were reviewed and approved by University of Malawi College of Medicine, Research Ethics Committee. Written informed consent to participate in this study was provided by the participants' legal guardian/next of kin.

## Author Contributions

MK and IC conceptualized the idea, wrote the first draft. ND and LH revised all the versions. All authors have read and approved the manuscript.

## Conflict of Interest

The authors declare that the research was conducted in the absence of any commercial or financial relationships that could be construed as a potential conflict of interest.

## Footnotes

^1^A percentile indicates the value below which a given percentage of observations in a group of observations fall.
