# Supplementary material for: Assessing the Impact of an Intervention Project by the Young women's Christian Association of Malawi on Psychosocial Well-Being of Adolescent Mothers and Their Children in Malawi
Source: Front Public Health. 2021 Mar 24;9:585517. doi: 10.3389/fpubh.2021.585517 (PMC8024460; doi:10.3389/fpubh.2021.585517)
Supplement: Supplementary file 1 [file Table_1.DOCX]

|  | **Baseline** | | | | | **End line** | | | | |
| --- | --- | --- | --- | --- | --- | --- | --- | --- | --- | --- |
|  | **N= 207** | | | | | **N= 211** | | | | |
| Brief Resilience Scale | Strongly Disagree | **Disagree** | Neutral | Agree | **Strongly Agree** | Strongly Disagree | **Disagree** | Neutral | Agree | **Strongly Agree** |
| I tend to bounce back quickly after hard times | 12 | **37** | 4 | 121 | **33** | 0 | **13** | 1 | 195 | **2** |
|  | 5.80% | **17.87%** | 1.93% | 58.45% | **15.94%** | 0.00% | **6.16%** | 0.47% | 92.42% | **0.95%** |
| I have a hard time making it through stressful events. | 24 | **47** | 4 | 110 | **22** | 0 | **43** | 2 | 165 | **1** |
|  | 11.59% | **22.71%** | 1.93% | 53.14% | **10.63%** | 0.00% | **20.38%** | 0.95% | 78.20% | **0.47%** |
| It does not take me long to recover from a stressful event. | 12 | **57** | 1 | 104 | **33** | 0 | **42** | 3 | 166 | **1** |
|  | 5.80% | **27.54%** | 0.48% | 50.24% | **15.94%** | 0.00% | **19.43%** | 1.42% | 78.67% | **0.47%** |
| It is hard for me to snap back when something bad happens | 17 | **49** | 5 | 112 | **24** | 0 | **60** | 1 | 150 | **0** |
|  | 8.21% | **23.67%** | 2.42% | 54.11% | **11.59%** | 0.00% | **28.44%** | 0.47% | 71.09% | **0.00%** |
| I usually come through difficult times with little trouble. | 23 | **57** | 6 | 100 | **21** | 0 | **50** | 1 | 160 | **0** |
|  | 11.11% | **27.54%** | 2.90% | 48.31% | **10.14%** | 0.00% | **23.70%** | 0.47% | 75.83% | **0.00%** |
| I tend to take a long time to get over setbacks in my life. | 21 | **49** | 5 | 111 | **21** | 0 | **61** | 0 | 149 | **1** |
|  | 10.14% | **23.67%** | 2.42% | 53.62% | **10.14%** | 0.00% | **28.91%** | 0.00% | 70.62% | **0.47%** |
| Average | 8.78% | **23.83%** | 2.01% | 52.98% | **12.40%** | 0.00% | **21.17%** | 0.63% | 77.81% | **0.39%** |

Supplementary Table 1 – Brief resilience scale
