# Supplementary material for: Assessing the Impact of an Intervention Project by the Young women's Christian Association of Malawi on Psychosocial Well-Being of Adolescent Mothers and Their Children in Malawi
Source: Front Public Health. 2021 Mar 24;9:585517. doi: 10.3389/fpubh.2021.585517 (PMC8024460; doi:10.3389/fpubh.2021.585517)
Supplement: Supplementary file 2 [file Table_2.DOCX]

|  | **Baseline** | | | | **Endline** | | | |
| --- | --- | --- | --- | --- | --- | --- | --- | --- |
|  | **N=207** | | | | **N=211** | | | |
| Rosenberg Self-Esteem Scale | Strongly Disagree | **Disagree** | Agree | **Strongly Agree** | Strongly Disagree | **Disagree** | Agree | **Strongly Agree** |
| On the whole, I am satisfied with myself. | 4 | **22** | 129 | **52** | 1 | **20** | 186 | **4** |
|  | 1.93% | **10.63%** | 62.32% | **25.12%** | 0.47% | **9.48%** | 88.15% | **1.90%** |
| At times I think I am no good at all. | 24 | **65** | 91 | **27** | 1 | **86** | 124 | **0** |
|  | 11.59% | **31.40%** | 43.96% | **13.04%** | 0.47% | **40.76%** | 58.77% | **0.00%** |
| I feel that I have a number of good qualities. | 10 | **20** | 138 | **39** | 1 | **14** | 196 | **0** |
|  | 4.83% | **9.66%** | 66.67% | **18.84%** | 0.47% | **6.64%** | 92.89% | **0.00%** |
| I am able to do things as well as most other people. | 8 | **27** | 123 | **49** | 1 | **20** | 188 | **2** |
|  | 3.86% | **13.04%** | 59.42% | **23.67%** | 0.47% | **9.48%** | 89.10% | **0.95%** |
| I feel I do not have much to be proud of. | 23 | **36** | 124 | **24** | 0 | **55** | 152 | **4** |
|  | 11.11% | **17.39%** | 59.90% | **11.59%** | 0.00% | **26.07%** | 72.04% | **1.90%** |
| I certainly feel useless at times. | 33 | **58** | 98 | **18** | 1 | **94** | 116 | **0** |
|  | 15.94% | **28.02%** | 47.34% | **8.70%** | 0.47% | **44.55%** | 54.98% | **0.00%** |
| I feel that I'm a person of worth, at least on an equal plane with others. | 7 | **36** | 117 | **47** | 0 | **16** | 192 | **3** |
|  | 3.38% | **17.39%** | 56.52% | **22.71%** | 0.00% | **7.58%** | 91.00% | **1.42%** |
| I wish I could have more respect for myself. | 7 | **-** | 154 | **46** | 2 | **7** | 202 | **0** |
|  | 3.38% |  | 74.40% | **22.22%** | 0.95% | **3.32%** | 95.73% | **0.00%** |
| All in all, I am inclined to feel that I am a failure. | 30 | **67** | 95 | **15** | 10 | **96** | 111 | **1** |
|  | 14.49% | **32.37%** | 45.89% | **7.25%** | 1.42% | **45.50%** | 52.61% | **0.47%** |
| I take a positive attitude toward myself. | 6 | **7** | 149 | **45** | 0 | **11** | 198 | **2** |
|  | 2.90% | **3.38%** | 71.98% | **21.74%** | 0.00% | **5.21%** | 93.84% | **0.95%** |

Supplementary Table 2 – Rosenberg self-esteem scale
