# Supplementary material for: Assessing the Impact of an Intervention Project by the Young women's Christian Association of Malawi on Psychosocial Well-Being of Adolescent Mothers and Their Children in Malawi
Source: Front Public Health. 2021 Mar 24;9:585517. doi: 10.3389/fpubh.2021.585517 (PMC8024460; doi:10.3389/fpubh.2021.585517)
Supplement: Supplementary file 3 [file Table_3.DOCX]

|  | **Baseline** | | | | | **Endline** | | | | |
| --- | --- | --- | --- | --- | --- | --- | --- | --- | --- | --- |
|  | **N=207** | | | | | **N=211** | | | | |
| **Parental Stress Scale** | **Strongly Disagree** | **Disagree** | **Undecided** | **Agree** | **Strongly Agree** | **Strongly Disagree** | **Disagree** | **Undecided** | **Agree** | **Strongly Agree** |
| I am happy in my role as a parent. | 10 | **13** | 2 | 135 | **47** | 2 | **7** | 2 | 198 | **2** |
|  | 4.83% | **6.28%** | 0.97% | 65.22% | **22.71%** | 0.95% | **3.32%** | 0.95% | 93.84% | **0.95%** |
| There is little or nothing I wouldn't do for my child(ren) if it was necessary. | 20 | **39** | 8 | 116 | **24** | 0 | **50** | 1 | 159 | **1** |
|  | 9.66% | **18.84%** | 3.86% | 56.04% | **11.59%** | 0.00% | **23.70%** | 0.47% | 75.36% | **0.47%** |
| Caring for my child(ren) sometimes takes more time and energy than I have to give. | 26 | **49** | 1 | 112 | **19** | 1 | **66** | 1 | 142 | **1** |
|  | 12.56% | **23.67%** | 0.48% | 54.11% | **9.18%** | 0.47% | **31.28%** | 0.47% | 67.30% | **0.47%** |
| I sometimes worry whether I am doing enough for my child(ren). | 22 | **31** | 2 | 128 | **24** | 0 | **45** | 1 | 164 | **1** |
|  | 10.63% | **14.98%** | 0.97% | 61.84% | **11.59%** | 0.00% | **21.33%** | 0.47% | 77.73% | **0.47%** |
| I feel close to my child(ren). | 3 | **2** | 1 | 154 | **47** | 0 | **3** | 2 | 205 | **1** |
|  | 1.45% | **0.97%** | 0.48% | 74.40% | **22.71%** | 0.00% | **1.42%** | 0.95% | 97.16% | **0.47%** |
| I enjoy spending time with my child(ren). | 5 | **4** | 2 | 152 | **44** | 0 | **1** | 0 | 207 | **3** |
|  | 2.42% | **1.93%** | 0.97% | 73.43% | **21.26%** | 0.00% | **0.47%** | 0.00% | 98.10% | **1.42%** |
| My child(ren) is an important source of affection for me. | 9 | **3** | 1 | 145 | **49** | 0 | **2** | 1 | 204 | **4** |
|  | 4.35% | **1.45%** | 0.48% | 70.05% | **23.67%** | 0.00% | **0.95%** | 0.00% | 96.68% | **1.90%** |
| Having child(ren) gives me a more certain and optimistic view for the future. | 7 | **7** | 3 | 147 | **43** | 0 | **9** | 0 | 197 | **5** |
|  | 3.39% | **3.39%** | 1.45% | 71.01% | **20.77%** | 0.00% | **4.27%** | 0.00% | 93.36% | **2.37%** |
| The major source of stress in my life is my child(ren). | 37 | **52** | 2 | 97 | **19** | 4 | **58** | 0 | 149 | **20** |
|  | 17.87% | **25.12%** | 0.97% | 46.86% | **9.18%** | 1.90% | **27.49%** | 0.00% | 70.62% | **8.06%** |
| Having child(ren) leaves little time and flexibility in my life. | 26 | **60** | 3 | 97 | **21** | 2 | **85** | 0 | 124 | **0** |
|  | 12.56% | **28.99%** | 1.45% | 46.86% | **10.14%** | 0.95% | **40.28%** | 0.00% | 58.77% | **0.00%** |
| Having child(ren) has been a financial burden. | 27 | **78** | 2 | 78 | **22** | 5 | **112** | 0 | 91 | **3** |
|  | 13.04% | **37.68%** | 0.97% | 37.68% | **10.63%** | 2.37% | **53.08%** | 0.00% | 43.13% | **1.42%** |
| It is difficult to balance different responsibilities because of my child(ren). | 25 | **68** | 2 | 94 | **18** | 0 | **98** | 0 | 112 | **1** |
|  | 12.08% | **32.85%** | 0.97% | 45.41% | **8.70%** | 0.00% | **46.45%** | 0.00% | 53.08% | **0.47%** |
| The behaviour of my child(ren) is often embarrassing or stressful to me. | 45 | **124** | 3 | 31 | **4** | 2 | **174** | 0 | 34 | **1** |
|  | 21.74% | **59.90%** | 1.45% | 14.98% | **1.93%** | 0.95% | **82.46%** | 0.00% | 16.11% | **0.47%** |
| If I had it to do over again, I might decide not to have child(ren). | 27 | **91** | 2 | 58 | **29** | 0 | **148** | 1 | 62 | **0** |
|  | 13.04% | **43.96%** | 0.97% | 28.02% | **14.01%** | 0.00% | **70.14%** | 0.47% | 29.38% | **0.00%** |
| I feel overwhelmed by the responsibility of being a parent. | 30 | **57** | 6 | 90 | **24** | 0 | **99** | 0 | 111 | **1** |
|  | 14.49% | **27.54%** | 2.90% | 43.48% | **11.59%** | 0.00% | **46.92%** | 0.00% | 52.61% | **0.47%** |
| Having child(ren) has meant having too few choices and too little control over my life. | 21 | **53** | 2 | 103 | **28** | 1 | **92** | 0 | 118 | **0** |
|  | 10.14% | **25.60%** | 0.97% | 49.76% | **13.58%** | 0.47% | **43.60%** | 0.00% | 55.92% | **0.00%** |
| I am satisfied as a parent. | 11 | **19** | 2 | 123 | **52** | 2 | **9** | 0 | 199 | **1** |
|  | 5.31% | **9.18%** | 0.97% | 59.42% | **25.12%** | 0.95% | **4.27%** | 0.00% | 94.31% | **0.47%** |
| I find my child(ren) enjoyable. | 4 | **1** | - | 143 | **59** | 2 | **2** | 0 | 200 | **7** |
|  | 1.93% | **0.48%** |  | 69.08% | **28.50%** | 0.95% | **0.95%** | 0.00% | 94.79% | **3.32%** |

Supplementary table 3 – Parental stress levels
