# Supplementary material for: Assessing the Impact of an Intervention Project by the Young women's Christian Association of Malawi on Psychosocial Well-Being of Adolescent Mothers and Their Children in Malawi
Source: Front Public Health. 2021 Mar 24;9:585517. doi: 10.3389/fpubh.2021.585517 (PMC8024460; doi:10.3389/fpubh.2021.585517)
Supplement: Supplementary file 4 [file Table_4.DOCX]

|  |  | **Baseline** | **Endline** |
| --- | --- | --- | --- |
| **Area of interaction** | **Assessment** | **n and % (N= 221)** | **n and % (N= 211)** |
| Predominant Maternal Mood/Affect | Flat, angry, or annoyed expression, spends little time looking at Infant, may read or engage in activities which divert attention from Infant | 41 | 5 |
|  |  | 18.55% | 2.37% |
|  | Sad expression, may appear preoccupied or distant, spends little time looking at Infant | 21 | 7 |
|  |  | 9.50% | 3.32% |
|  | Tense, anxious expression, easily distractible; Mother may appear worried and/or annoyed alternating with occasional expressions of pleasure | 28 | 7 |
|  |  | 12.67% | 3.32% |
|  | Bland expression, at times disinterested or distracted occasionally smiles at Infant and gazes at Infant for 5-10 second intervals | 48 | 17 |
|  |  | 21.72% | 8.06% |
|  | Shifting expression of pleasure or tenderness; shifts expression in response to Infant behaviour, prolonged visual regard, smiles at Infant. Mother's attention may be briefly distracted by events in room, but Mother's overall focus is on Infant | 83 | 175 |
|  |  | 37.56% | 82.94% |
| Maternal Verbalization (Content) | Speech to Infant is strongly critical and or derogatory in content | 11 | 5 |
|  |  | 4.98% | 2.37% |
|  | Speech to Infant is disapproving in content, expressing dissatisfaction with something Infant has done, or lnfant's appearance | 20 | 1 |
|  |  | 9.05% | 0.47% |
|  | Speech to Infant is neutral in content, does not specifically express approval or disapproval | 36 | 14 |
|  |  | 16.29% | 6.64% |
|  | Speech to Infant is affectionate in content, expresses approval | 79 | 30 |
|  |  | 35.75% | 14.22% |
|  | Speech to Infant is warmly affectionate and loving in content expressing extremely positive attributes (e.g., I love you; you're such a beautiful baby) | 75 | 161 |
|  |  | 33.94% | 76.30% |
| Maternal Modulation Of Distress Episodes | Prolonged delay, allows Infant to reach state of prolonged distress before intervention | 15 | 2 |
|  |  | 6.79% | 0.95% |
|  | Long delay, allows Infant to reach full blown crying state before intervening | 8 | 1 |
|  |  | 3.62% | 0.47% |
|  | Delays, allows Infant to reach distress state, crying for brief period before intervening | 11 | 4 |
|  |  | 4.98% | 1.90% |
|  | Delays somewhat, allows fretting and/or fussing before intervening. Score even if occurs only once | 25 | 14 |
|  |  | 11.31% | 6.64% |
|  | Mother prevents distress episode by noticing beginning signs or subtle cues (e.g., pre-cry grimaces) and intervenes before Infant reaches a clear distress episode | 79 | 37 |
|  |  | 35.75% | 17.54% |
|  | No distress during observation period | 83 | 153 |
|  |  | 37.56% | 72.51% |
| Predominant Infant Mood/Affect | Withdrawn, inactive, depressed, may shut out stimuli by sleep like behaviour | 7 | 4 |
|  |  | 3.17% | 1.90% |
|  | Restless, tense, irritable | 17 | 6 |
|  |  | 7.69% | 2.84% |
|  | Tolerant, bland, placid, disinterested, alternating with periods of tension and restlessness | 44 | 16 |
|  |  | 19.91% | 7.58% |
|  | Pleasant, relaxed, appears interested | 102 | 59 |
|  |  | 46.15% | 27.96% |
|  | Pleasurable expression with periods of brightening, smiling | 51 | 126 |
|  |  | 23.08% | 59.72% |
| Synchrony In Response to Pleasurable Affect** | No pleasurable exchange | 44 | 7 |
|  |  | 19.91% | 3.32% |
|  | Infant enters state of excitement and/or pleasure and Mother does not respond | 17 | 9 |
|  |  | 7.69% | 4.27% |
|  | Infant enters state of excitement and/or pleasure and Mother abruptly interferes resulting in shift in I mood (e.g., shutdown, distress) | 18 | 9 |
|  |  | 8.14% | 4.27% |
|  | Infant enters state of excitement and/or pleasure, Mother delays then responds to Infant appropriately with verbalization, gestures, and sharing excitement | 58 | 20 |
|  |  | 26.24% | 9.48% |
|  | Mother and Infant enter into pleasurable exchange almost simultaneously | 84 | 169 |
|  |  | 38.01% | 80.09% |

Supplementary table 4: Mother-infant interaction
