# Supplementary material for: Assessing the Impact of an Intervention Project by the Young women's Christian Association of Malawi on Psychosocial Well-Being of Adolescent Mothers and Their Children in Malawi
Source: Front Public Health. 2021 Mar 24;9:585517. doi: 10.3389/fpubh.2021.585517 (PMC8024460; doi:10.3389/fpubh.2021.585517)
Supplement: Supplementary file 5 [file Table_5.DOCX]

|  | **Baseline** | | | |  |  | **Endline** | | | |
| --- | --- | --- | --- | --- | --- | --- | --- | --- | --- | --- |
| **My partner(s):** | **Yes it happened to them (N varied)** | | **Yes the child witnessed it (N varied)** | |  |  | **Yes it happened to them (N varied)** | | **Yes the child witnessed it (N varied)** | |
|  | **n** | **%** | **n** | **%** |  |  | **n** | **%** | **n** | **%** |
| Blamed me for causing their violent behaviour | 56 | **21.05%** | 21 | **8.50%** |  |  | 21 | **9.95%** | 11 | **5.79%** |
| Shook, pushed, grabbed or threw me | 45 | **17.05%** | 20 | **8.03%** |  |  | 25 | **11.85%** | 15 | **7.85%** |
| Tried to convince my family, children or friends that I am crazy or tried to turn them against me | 38 | **14.50%** | 20 | **7.94%** |  |  | 21 | **9.95%** | 14 | **7.73%** |
| Used or threatened to use a knife or gun or other weapon to harm me | 25 | **9.51%** | 13 | **5.20%** |  |  | 3 | **3.79%** | 6 | **3.79%** |
| Made me perform sex acts that I did not want to perform. | 45 | **17.11%** | 17 | **6.85%** |  |  | 10 | **10.95%** | 6 | **3.11%** |
| Followed me or hung around outside my home or work. | 42 | **15.85%** | 15 | **6.07%** |  |  | 19 | **9.05%** | 10 | **5.21%** |
| Threatened to harm or kill me or someone close to me | 28 | **10.65%** | 16 | **6.50%** |  |  | 11 | **5.21%** | 5 | **2.66%** |
| Choked me. | 16 | **6.08%** | 4 | **1.62%** |  |  | 10 | **4.74%** | 6 | **3.17%** |
| Forced or tried to force me to have sex. | 32 | **11.99%** | 14 | **5.74%** |  |  | 25 | **11.85%** | 6 | **3.13%** |
| Harassed me by phone, text, email or using social media. | 41 | **15.36%** | 16 | **6.48%** |  |  | 38 | **18.10%** | 9 | **4.69%** |
| Told me I was crazy, stupid or not good enough. | 65 | **24.53%** | 32 | **12.75%** |  |  | 25 | **11.85%** | 14 | **7.41%** |
| Hit me with a fist or object, kicked or bit me. | 36 | **14.66%** | 19 | **7.76%** |  |  | 15 | **7.21%** | 8 | **4.26%** |
| Kept me from seeing or talking to my family or friends. | 36 | **13.58%** | 19 | **7.66%** |  |  | 19 | **9.05%** | 11 | **5.79%** |
| Confined or locked me in a room or other space. | 17 | **6.42%** | 9 | **3.77%** |  |  | 5 | **2.37%** | 1 | **0.53%** |
| Kept me from having access to a job, money or financial resources. | 34 | **13.03%** | 10 | **4.08%** |  |  | 27 | **12.80%** | 14 | **7.33%** |

Supplementary table 5: Intimate Partner Violence
